# Lung cancer incidence decreases with elevation: evidence for oxygen as an inhaled carcinogen

**DOI:** 10.7717/peerj.705

**Published:** 2015-01-13

**Authors:** Kamen P. Simeonov, Daniel S. Himmelstein

**Affiliations:** 1 Perelman School of Medicine, University of Pennsylvania, Philadelphia, PA, USA; 2 Biological & Medical Informatics, University of California, San Francisco, CA, USA

**Keywords:** Cancer incidence, Lung cancer, Altitude, Elevation, Oxygen

## Abstract

The level of atmospheric oxygen, a driver of free radical damage and tumorigenesis, decreases sharply with rising elevation. To understand whether ambient oxygen plays a role in human carcinogenesis, we characterized age-adjusted cancer incidence (compiled by the National Cancer Institute from 2005 to 2009) across counties of the elevation-varying Western United States and compared trends displayed by respiratory cancer (lung) and non-respiratory cancers (breast, colorectal, and prostate). To adjust for important demographic and cancer-risk factors, 8–12 covariates were considered for each cancer. We produced regression models that captured known risks. Models demonstrated that elevation is strongly, negatively associated with lung cancer incidence (*p* < 10^−16^), but not with the incidence of non-respiratory cancers. For every 1,000 m rise in elevation, lung cancer incidence decreased by 7.23 99% CI [5.18–9.29] cases per 100,000 individuals, equivalent to 12.7% of the mean incidence, 56.8. As a predictor of lung cancer incidence, elevation was second only to smoking prevalence in terms of significance and effect size. Furthermore, no evidence of ecological fallacy or of confounding arising from evaluated factors was detected: the lung cancer association was robust to varying regression models, county stratification, and population subgrouping; additionally seven environmental correlates of elevation, such as exposure to sunlight and fine particulate matter, could not capture the association. Overall, our findings suggest the presence of an inhaled carcinogen inherently and inversely tied to elevation, offering epidemiological support for oxygen-driven tumorigenesis. Finally, highlighting the need to consider elevation in studies of lung cancer, we demonstrated that previously reported inverse lung cancer associations with radon and UVB became insignificant after accounting for elevation.

## Introduction

At present, four types of cancer—prostate, breast, lung, and colorectal—exceed 100,000 new cases per year in the United States. Of these cancers, lung cancer carries the worst prognosis and will claim an estimated 159,260 lives in 2014 (Siegel et al., 2014). While lung cancer primarily afflicts smokers, 10–15% of cases arise in nonsmokers (Samet et al., 2009), and over 80% of smokers never develop lung cancer (Bilello, Murin & Matthay, 2002, p. 5). Additional characterized risk factors include genetic susceptibility as well as environmental exposure to carcinogens such as radon, asbestos, and fine-particulate matter (Subramanian & Govindan, 2007). This multifactorial etiology for lung cancer could include long-term exposure to an inhaled carcinogen.

Inspired molecular oxygen (O_2_) leads to intracellular formation of reactive oxygen species (ROS). This occurs either by spontaneous ionizing radiation or by incomplete reduction of O_2_ during normal cellular respiration (Fridovich, 1988). ROS are highly unstable and undergo damaging redox reactions with a range of cellular components (Jackson, 1985). A variety of antioxidant enzymes and pathways exist to eliminate ROS (Matés, Pérez-Gómez & Núñez de Castro, 1999). However, formation and elimination of ROS is a stochastic process during which cells accumulate damage, including mutations from reactions with nucleic acids (Cooke et al., 2003).

The amount of DNA damage and cytotoxicity incurred is influenced both by the effectiveness of oxygen metabolism (Passos et al., 2007; Sung et al., 2010) and the extent of oxygen exposure (Bruyninckx, Mason & Morse, 1978; Packer & Fuehr, 1977; Parrinello et al., 2003). Oxidative DNA damage plays a prominent role in the pathogenesis and exacerbation of many diseases including cancer (Cooke et al., 2003). A recent study of cancer initiation in three mouse models of tumorigenesis—*P*53^(−/−)^, *APC*^(*min*/+)^, and a chemically-induced model—found that halving ambient oxygen exposure led to proportional increases in tumor-free survival time and decreases in genomic instability and tumor bulk (Sung et al., 2011). While similar studies are impossible in humans, numerous reports have indicated significant increases in childhood cancers in cases of neonatal oxygen supplementation (Maruyama et al., 2000; Naumburg et al., 2002; Oue et al., 2003; Spector et al., 2005). Importantly, oxygen toxicity appears most profound in the lung, where exposure is direct (Jackson, 1985; Nagato et al., 2012; Pagano & Barazzone-Argiroffo, 2003).

Despite the inability to perform controlled experiments of oxygen toxicity in a human setting, elevation provides a natural experimental platform for examining the effects of oxygen on carcinogenesis. The relation between elevation and barometric pressure, and hence oxygen, is roughly linear at habitable altitudes. Across United States counties, elevation accounts for a 34.9% decrease in oxygen from Imperial County, California (−11 m) to San Juan County, Colorado (3,473 m). From its partial pressure at sea level, oxygen is reduced to 88.7% at 1,000 m, 78.5% at 2,000 m, and 69.2% at 3,000 m (Berberan-Santos, 1997). Taking advantage of this natural dosage gradient, we asked whether atmospheric oxygen, assessed via elevation, associates with carcinogenesis.

Numerous reports and observations of lower cancer rates at higher elevations appear in the literature of the last four decades (Amsel et al., 1982; Burton, 1975; Hayes, 2010; Mason & Miller, 1974; Van Pelt, 2003; Weinberg, Brown & Hoel, 1987). Of particular relevance, Weinberg, Brown & Hoel (1987) and Van Pelt (2003) suggest reduced oxygen as a possible explanation. Interestingly, both studies investigate elevation as a confounder of radiation hormesis—the theory that low, environmental doses of radiation are protective against cancer. Inevitably, neither study was designed to specifically assess elevation, particularly how its effect on atmospheric pressure relates to cancer. Weinberg, Brown & Hoel (1987) focused on a small sample of 80 metropolitan areas without a systematic selection process, while only adjusting for proxies of urbanization and ethnicity without accounting for other demographic or risk factors such as smoking. Regarding Van Pelt (2003), county elevation exposure was estimated by the elevation of the largest city, rather than a more precise population-weighted calculation. Adjustment for potential confounders was limited to subgrouping by sex and correction for smoking prevalence. However, statewide smoking prevalence was uniformly applied to all counties within a state. Moreover, both studies examined cancer mortality instead of the more direct outcome of incidence. All of these issues contribute to a limited ability to compare effects across different cancer sites (i.e., respiratory versus non-respiratory sites). While much was unconsidered due to each group’s interest in elevation primarily as a confounder, many of these issues were simply due to a lack of available data. Elevation profoundly impacts variables ranging from climate to behavior (Burtscher, 2014). To isolate the atmospheric-based effects of elevation on cancer incidence, many factors must be carefully considered. A nuanced analysis with precise, high-resolution data is required.

Building on existing experimental and epidemiological evidence, we designed a study to assess the effect of elevation-dependent ambient oxygen on cancer incidence. We focused on the elevation-varying western United States, maximizing variation in our exposure of interest while minimizing potential confounding. Recent proliferation of high-resolution, publicly-available data enabled a precise ecological evaluation of our hypothesis. We relied on county-level incidence rather than mortality to minimize quality of care and disease progression biases. To accurately assess oxygen exposure, we incorporated subcounty population dispersion into county elevation calculation. We accounted for potential confounding effects by including important risk and demographic factors and evaluating a range of environmental variables that covary with elevation. We compared the association of elevation with lung cancer versus its association with breast, colon, and prostate cancers to discriminate between atmosphere dependent and independent elevation effects. These steps combined with a robust and conservative statistical framework provided a rigorous assessment of our hypothesis: cancer incidence decreases as elevation rises, a trend most pronounced in tissue with direct atmospheric exposure.

## Methods

### Data collection & preparation

From 11 publicly-available databases, we compiled US county data on cancer risk factors, environmental features, demographics, and quality control metrics, while avoiding redundancy. When selecting resources, we balanced several considerations including coverage, precision, collection period, and accessibility. To capture the long latency period of cancers and minimize observational error, we preferred collection periods preceding the cancer incidence timeframe and spanning multiple years. Resources were integrated using county FIPS (Federal Information Processing Standards) codes if available and name matching otherwise. Variables collected as cancer incidence predictors are displayed in Table 1. Variables were averaged over their entire collection periods. Unless otherwise noted, averaging was performed by the source databases.

**Table 1:** Predictor information and inclusion. The cancers that each predictor was included for are denoted by ‘l’ (lung), ‘b’ (breast), ‘c’ (colorectal), ‘p’ (prostate), ‘all’ (all 4 cancers), and ‘env’ (as an elevation replacement in the environmental analysis). The data collection period, number of counties with non-missing values after quality control, and unweighted mean and standard deviation are also reported.

Predictor	Cancers	*n*	Mean	sd	Years	Units
Black	all	259	1.9	2.6	2000	%
Education	all	259	24	10	2006–2010	% of adults with bachelor’s
Income	all	259	49	11	2006–2010	thousands of US $
Metro	all	259	0.43	0.5	2003	binary classification
Obesity	all	259	21	3.5	2003–2005	% prevalence
White	all	259	86	11	2000	%
Elevation	all	259	0.97	0.74	2000	kilometers
Diabetes	c	259	6.6	1	2004–2008	% age-adjusted prevalence
Drinking	b, c	244	15	3.8	2002–2008	% binge drinking last 30 days
Female smoking	b	258	41	6.8	1997–2003	% smoked in lifetime
Male	c, l	259	50	1.6	2000	%
Mammogram	b	259	64	6.2	2000–2003	% within last two years
Meat	c, p	259	67	12	2006	lbs per household per year
Other cancer	b	259	273	33	2005–2009	age-adjusted incidence per 100,000
Other cancer	c	258	396	42	2005–2009	age-adjusted incidence per 100,000
Other cancer	l	255	382	36	2005–2009	age-adjusted incidence per 100,000
Other cancer	p	259	352	48	2005–2009	age-adjusted incidence per 100,000
Smoking	l, c	258	47	5.8	1997–2003	% smoked in lifetime
Particulate	l, env	259	10	1.8	2003–2008	µg/m^3^
Radon	l, env	258	1.7	1		picocuries per liter
UVB	env	259	1,072	239	1996–2005	kJ/m^2^
Sunlight	env	259	17,060	1,939	1979–2000	kJ/m^3^
Precipitation	env	259	1.9	1.5	1979–2000	average daily mm
High temp	env	259	16	4.6	1979–2000	°C
Diurnal temp	env	259	9.5	1.6	1979–2000	°C

#### Cancer incidence

Total county cancer incidences (‘All Races (incl. Hisp)’, ‘Both Sexes’, ‘All Ages’) were obtained from the National Cancer Institute (NCI) State Cancer Profiles for the following categories: ‘Lung & Bronchus’, ‘Breast’, ‘Prostate’, ‘Colon & Rectum’, and ‘All Cancer Sites’ (National Cancer Institute, 2013b). For ‘All Cancer Sites’, incidence for ‘Sex Males’ and ‘Sex Females’ was downloaded. ‘Lung & Bronchus’ incidence was obtained for ‘Sex Males’, ‘Sex Females’, ‘Age 65+’, and ‘Age <65’. The data was collected from 2005 to 2009, age-adjusted to the 2000 US standard population, and converted to cases per 100,000 individuals per year. For each cancer, we calculated the incidence for ‘other cancer’ by subtracting the relevant cancer’s incidence from the incidence for all sites combined. Breast and prostate incidences were subtracted from the corresponding sex-specific all-sites incidences.

#### Demographic & health data

County-level education, income, and mammogram data were obtained from State Cancer Profiles (National Cancer Institute, 2013b), which derived data as follows: median household income and percent of individuals over 25 with a bachelor’s degree were calculated from the American Community Survey data spanning 2006–2010; the percentage of women over 40 who received a mammogram in the past two years was calculated from the Behavioral Risk Factor Surveillance System (BRFSS) and the National Health Interview Survey (NHIS) for the period 2000–2003.

The percent of individuals having reported smoking over 100 cigarettes in their lifetime was downloaded from NCI Small Area Estimates (National Cancer Institute, 2013a). This resource provides model-based lifetime smoking estimates from BRFSS and NHIS data for the periods 1997–1999 and 2000–2003 (Raghunathan et al., 2007). We downloaded separate estimates for males, females, and all individuals and averaged the estimates over the two time periods. The percent of adults that reported binge drinking in the past 30 days, calculated from BRFSS data spanning 2002–2008, was obtained from the County Health Rankings 2010 release (University of Wisconsin Population Health Institute, 2014). Pounds of meat purchased per household during 2006 was extracted from the 2011 Food Environment Atlas (United States Department of Agriculture Economic Research Service, 2014).

Age-adjusted model-based estimates for obesity and diabetes prevalence were downloaded from the Centers for Disease Control (CDC) (Centers for Disease Control and Prevention, 2013b). The estimates were calculated from BRFSS data spanning 2004–2008 for diabetes and 2003–2005 for obesity. Diabetes was reported annually, so we averaged its prevalence over the five available years. Obesity refers to the percent of individuals over 20 who reported a body mass index of 30 or higher.

A classification of counties as metropolitan or nonmetropolitan produced in 2003 was obtained from the USDA Economic Research Service (United States Department of Agriculture Economic Research Service , 2013). County race, population, and migration information was downloaded from the US Census Bureau for the 2000 census (United States Census Bureau, 2013). To achieve subcounty data resolution, county blockgroup boundaries and populations were also downloaded from the Census Bureau. The percents white, black, and Native American for counties were determined by the Census as the percent of individuals claiming that race alone or in combination with another race. Census blockgroup boundaries and the corresponding populations were downloaded in the TIGER shapefile format. We calculated 5-year county immigration rates by dividing the difference between total movers and within-county movers by the total population. Percent male was calculated using 2000-census data prepared by the National Atlas (National Atlas of the United States, 2004).

#### Climatic & environmental data

County averages for minimum and maximum daily temperature (°C), fine particulate matter with an aerodynamic diameter less than 2.5 micrometers (µg/m^3^), precipitation (mm), and sunlight (kJ/m^3^) were downloaded from the CDC WONDER database (Centers for Disease Control and Prevention, 2013a). The maximum data collection time intervals were specified: temperature, precipitation, and sunlight measurements were collected from 1979 to 2000, while fine particulate matter was collected from 2003 to 2008. We calculated average diurnal temperature variation by subtracting the average daily minimum temperature from the average daily maximum. Solar UVB exposure (kJ/m^2^), erythemally weighted to correspond to vitamin-D induction, was obtained from a 2006 study (Boscoe & Schymura, 2006). Indoor radon concentrations in picocuries per liter were obtained from the Lawrence Berkeley National Laboratory High-Radon Project (Lawrence Berkeley National Laboratory, 2013). Their model-based approach explained 64% of variation in mean radon concentration across 5027 living-areas (Apte, Nero & Revzan, 1998). Elevation data was downloaded from WorldClim at 30 arc-seconds (1 km) resolution (Hijmans et al., 2005; WorldClim, 2013).

#### Population-weighted mean elevation

Absolute barometric pressure provides a more direct measure of oxygen than does elevation, albeit marginally. However, barometric pressure data is collected at land stations, which are not universally distributed, and frequently reported relative to sea level for weather forecasting. Therefore, we relied on population-weighted elevation to assess atmospheric oxygen exposure.

We calculated county elevation by subdividing a county into census blockgroups, computing the mean elevation for each blockgroup, and calculating the population-weighted average of the blockgroup elevations. On average, US counties contained 66.5 blockgroups with an average population of 1348.3 persons per blockgroup. By accounting for population dispersion within counties, this method better assesses the inhabitants’ exposure to elevation than population agnostic methods. With greater computational resources, future researchers may choose to use census blocks as a finer subdivision combined with higher resolution elevation data. This framework can be generalized for measuring exposure to any topological variable across a geographical area.

### County filtering

We restricted the analysis to states in the contiguous United States with elevation spans exceeding 3,000 m. The selected states—AZ, CA, CO, ID, MT, NV, NM, OR, UT, WA, WY—contained 414 counties composing the Western United States. Next, counties were filtered for quality control. Counties with populations below 10,000 were excluded due to high missingness (values were missing for many of the variables) and observational error (values were present but subject to large margins of error, evidenced by source-reported confidence intervals). Counties with high Native American composition or immigration rates were considered potentially problematic: cancer rates among Native Americans are prone to misestimation (Puukka, Stehr-Green & Becker, 2005); and immigrants accumulate cancer risk prior to migration, outside of their destination county. Accordingly, we found that predictions of all-site cancer incidence, based on eight general demographic and health predictors, diverged from reported incidence for Native American and immigration-rich counties (Fig. S1). Selecting exclusion thresholds corresponding to this divergence, we omitted counties with five-year immigration rates exceeding 40% or Native American population exceeding 25%. After filtering, 260 counties remained.

### Regression analysis

We evaluated the association between elevation and cancer incidence using multivariate linear regression. Counties were weighted by their population square root up to a maximum population of 250,000 where measurement uncertainty leveled off to minimal levels. The weighting scheme accounted for increasing measurement uncertainty among low population counties without granting heavily populated counties an overwhelming influence.

To minimize confounding effects, we selected well-established factors to include as covariates with elevation. We avoided excessive collinearity (Dormann et al., 2013) by carefully identifying major cancer-specific risk factors with available county-level estimates. Smoking, radon, fine particulate matter, and percent male were identified for lung cancer; female smoking, mammogram, and drinking for breast cancer; smoking, drinking, diabetes, meat consumption, and percent male for colorectal cancer; and meat consumption for prostate cancer. We included six additional covariates—metro, white, black, education, income, and obesity—for all cancers to indirectly account for unknown or immeasurable risk factors or biases. Since a large degree of risk is shared between cancers (Danaei et al., 2005), for each cancer we included the incidence of all other cancer. In addition to elevation, a total of 11 covariates were included for lung, 10 for breast, 12 for colorectal, and 8 for prostate (Table 1). We created cancer-specific datasets by removing counties with any missing data for included variables. Standardized versions of each dataset were created by converting cancer incidence and all predictors to weighted *z*-scores.

**Figure 1:**
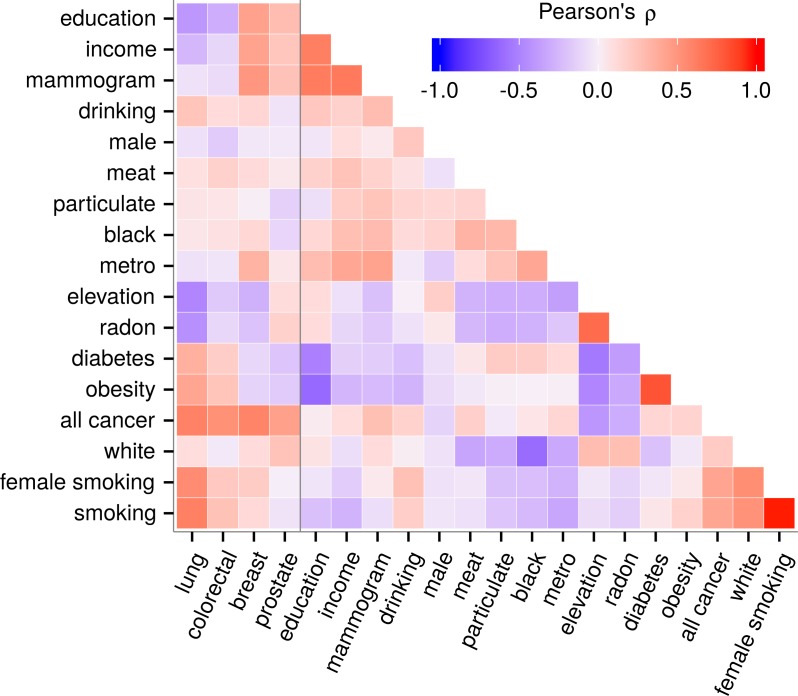
Predictor collinearity and correlation with cancer incidence. Predictors displayed expected correlations such as a strong positive correlation between obesity and diabetes. Collinearity was moderate but pervasive. Elevation covaried with most variables including cancers indicating the need to adjust for covariates while carefully considering collinearity. Besides radon, the correlation of elevation with other predictors did not exceed |*ρ*| = 0.55.

We employed two regression methods, best subset and lasso, in parallel. The best subset approach allowed us to force elevation into the model and exhaustively evaluate all possible models while remaining amenable to statistical interpretation. However, evaluating all possible subsets creates the potential for overfitting. Lasso addresses this concern by introducing coefficient shrinkage and variable selection (Tibshirani, 1996). Despite efforts to exclude redundant predictors, variables were characterized by moderate yet pervasive collinearity (Fig. 1). In the best subset method, severe collinearity could cause unstable and unreliable coefficient estimates with inflated standard errors. In addition to scrutinizing best subset results, we adopted the lasso, whose variable selection mechanism tends to include a single member from a group of correlated predictors. This characteristic makes the lasso effective at identifying truly associated predictors in the presence of high collinearity (Dormann et al., 2013).

#### Best subset regression

For each cancer, we performed best subset regression by evaluating all predictor subsets that included elevation. Subset sizes ranged from one, where elevation was the sole predictor, up to the total number of included variables. For a given size, the predictor set minimizing the residual weighted sum of squares was computed. To identify an optimal model across subset sizes, we chose the subset whose model minimized the Bayesian Information Criterion (BIC) (Schwarz, 1978). The BIC aims to balance the competing objectives of model parsimony and goodness of fit. Compared to alternative criteria, the BIC more harshly penalizes complexity, which is favorable in situations where extra terms risk exacerbating the effects of collinearity. To assess whether elevation was negatively correlated with incidence for each cancer, we applied a one-tailed *t*-test to the elevation coefficient in the optimal best subset model. A Bonferroni-adjusted significance cutoff of *p* = 0.0125 was adopted corresponding to a familywise error rate threshold of 5%.

#### Lasso regression

We fit a single model for each cancer using lasso regression (Tibshirani, 1996). Lasso requires a single regularization parameter. We optimized this parameter separately for each cancer using 10-fold cross-validation. To prevent overfitting, we adopted the ‘one-standard-error’ rule for determining the optimal parameter value (Friedman, Hastie & Tibshirani, 2010).

#### Partial regression plots

To display the relationship between elevation and cancer incidence while accounting for the effect of covariates, we employed partial regression plots. The *x*-axis represents the residual from regressing elevation against the remaining covariates. The *y*-axis represents the residual from regressing cancer incidence versus the included covariates absent elevation. The partial regression refers to the simple weighted regression of the cancer incidence residuals (*y*-axis) against the elevation residuals (*x*-axis). Underlying elevation and incidence values for each county are not discernable in the partial regression plots. However, the slope of the partial regression equals the multivariate elevation coefficient, and the residuals along the partial regression line are equivalent to the multivariate regression residuals. The partial coefficient of determination for elevation, computed as the partial *R*^2^, signifies the proportion of cancer variance explained by elevation.

### County stratifications

To investigate the potential of a smoking-elevation interaction affecting lung cancer, we partitioned counties into smoking prevalence terciles as follows: high-smoking (49.9–61.9), mid-smoking (44.9–49.9), low-smoking (28.2–44.9). Within each tercile, we regressed lung cancer incidence against elevation for visual examination. For statistical evaluation, we refitted the optimal best subset model with an added interaction term (standardized smoking prevalence × standardized elevation).

To mitigate uncontrolled confounding (i.e., omitted-variable bias), we stratified the lung cancer dataset by state. Health policy and data collection are often enacted at the state level, making stratification by state a sensible choice for maximizing within-strata homogeneity. Within each stratum, lung cancer incidence was regressed against elevation and smoking prevalence. The elevation effect size was estimated across the eleven state-specific models using a fixed-effects meta-analysis, which averaged elevation coefficients weighted by their inverse variances.

### Population subgroupings

We evaluated the association between elevation and lung cancer incidence measured for the following population subgroups: under 65 years old, 65 or older, males, and females. These subgroups were chosen because exposure to risk factors, such as occupational or lifestyle hazards, often segregates by sex or age. For this analysis, we created a dataset with counties that had no missing data for the four subgroup outcomes and the predictors from the optimal best subset model. Proceeding with the best subset covariates, we fit a separate regression model for each population subgroup. We used sex-specific smoking prevalence for the male and female models.

### Elevation substitutions

Environmental variables without established cancer risk were not evaluated in the previous regression analyses to avoid problems of collinearity. For lung and breast cancer, we investigated whether substituting elevation with each of seven environmental variables produced a more likely model, which could indicate an indirect elevation-cancer association. For each environmental variable, we performed best subset regression to find the BIC-minimizing set of predictors. The covariates evaluated for each cancer matched those from before (Table 1) with the exception of radon and fine particulate, which were excluded as lung cancer covariates and instead included as elevation replacements. For both cancers, the increase in minimum-BIC for each substitution compared to elevation was recorded. Change in BIC was converted to a Bayes factor, $K\approx {e}^{-\frac{1}{2}(\Delta B I C)}$ (Raftery, 1995, p. 139). *K* > 1 provides evidence favoring replacement whereas *K* < 1 provides evidence against.

### Software

Analyses were performed using the statistical-computing language *R*. County elevation computation relied on the GIS packages *raster* and *rgdal*. The best-subset regression analysis used the *leaps* package, which efficiently identifies top performing models from the complete search space. The *glmnet* package implemented the lasso (Friedman, Hastie & Tibshirani, 2010). The state-specific lung cancer elevation coefficients were meta-analyzed using the *metafor* package (Viechtbauer, 2010). Tables were exported using the *Hmisc* package. Plots were created with the *ggplot2* package. Correlation plots were ordered using Ward’s hierarchical clustering.

### Data availability

The county-level dataset compiled for this study is available (Data S1). The project GitHub repository (https://github.com/dhimmel/elevcan) contains the code used to perform analyses as well as all intermediate files.

## Results

### Strong, negative association between elevation & lung cancer incidence

Performing best subset regression for each cancer, we found a highly significant, strong negative association between elevation and lung cancer incidence with a standardized coefficient (*β_z_*) of −0.35 99% CI [−0.46, −0.25] (*p* < 10^−16^, one-tailed *t*-test) (Table 2). Lung cancer incidence decreased by 7.23 [5.67–8.80] cases (per 100,000 individuals) per kilometer rise in elevation, equating to 12.7% [9.1%–16.4%] of the mean lung cancer incidence. For other cancers, we found a weak, negative association with breast cancer (*β_z_* = −0.15, *p* < 10^−2^) but not with colorectal (*p* = 0.88) or prostate (*p* = 0.97) cancer.

**Table 2:** Summary of the optimal best subset model for each cancer. The weighted mean and standard deviation for each cancer incidence is reported. For each optimal best subset model, the number of counties (*n*) and predictors (size) as well as the *R*^2^ is indicated. The elevation *p*-value (one-tailed test for coefficient negativity) is denoted along with three versions of the elevation coefficient: unstandardized (*β*), standardized (*β_z_*), and as a percentage of mean incidence (*β*_%_).

Incidence		Model			Elevation			
Cancer	Mean (sd)	*n*	Size	*R* ^2^	*p*-value	*β*	*β_z_*	*β*_%_
Lung	56.8 (14.4)	253	5	70.2%	1.34× 10^−17^	−7.23	−0.35	−12.7%
						[−9.29, −5.18]	[−0.46, −0.25]	[−16.35, −9.11]%
Breast	119.3 (16.7)	243	6	56.8%	3.22× 10^−03^	−3.63	−0.15	−3.0%
						[−7.06, −0.20]	[−0.30, −0.01]	[−5.92, −0.17]%
Colorectal	41.9 (6.0)	243	5	34.1%	0.883	0.65	0.08	1.5%
						[−0.76, 2.06]	[−0.09, 0.24]	[−1.82, 4.92]%
Prostate	148.6 (23.9)	259	4	18.9%	0.974	4.71	0.14	3.2%
						[−1.56, 10.97]	[−0.05, 0.33]	[−1.05, 7.38]%

**Figure 2:**
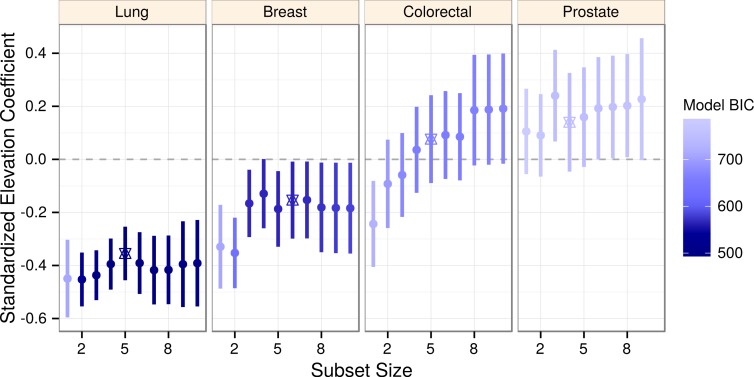
Elevation negatively associates with lung cancer incidence across a range of models. Elevation coefficients and 99% confidence intervals are plotted across a range of best subset model sizes for each cancer. As determined by BIC, more likely models are shaded darker. The optimal (BIC-minimizing) model for each cancer is denoted by a star. Lung cancer is the only cancer to display consistently negative coefficients and confidence intervals.

The optimal (BIC-minimizing) models contained five predictors for lung and colorectal cancers, six predictors for breast, and four predictors for prostate cancer (Table 2). Within each cancer, we compared the elevation coefficients across a range of model sizes (Fig. 2). Unique to lung cancer, elevation confidence intervals were consistent and wholly negative, indicating robustness to collinearity as well as to confounding by included covariates. Other cancers displayed greater coefficient variability and uncertainty, possibly due to covariate collinearity with elevation, which led us to implement lasso regression.

Lasso regression performs variable selection that operates well under moderate collinearity and coefficient shrinkage that prevents overfitting. Using a conservative setup of the lasso, we again observed a strong, negative association between elevation and lung cancer incidence with a standardized coefficient of −0.33, changing minimally from the best subset estimate (*β_z_* = −0.35) despite the strong regularization of the lasso (Table 3). For breast cancer, where the best subset model yielded an elevation coefficient with high uncertainty, the lasso reduced the estimate to a trivial level (*β_z_* = −0.02) indicating that overfitting contributed to best subset negativity. Meanwhile, the elevation term was absent in the colorectal and prostate lasso models. Together the regression methods indicated a negative association with elevation that was unique to lung cancer in terms of strength, significance, and statistical robustness.

**Table 3:** Summary of lasso models for each cancer. The number of predictors (size) and *R*^2^ for each cancer’s lasso model are reported. The corresponding elevation coefficients are displayed as unstandardized (*β*), standardized (*β_z_*), and as a percentage of mean incidence (*β*_%_). Refer to Table 2 for cancer-specific dataset information including county number and mean incidence.

Cancer	Size	*R* ^2^	*β*	*β_z_*	*β*_%_
Lung	6	67.1%	−6.64	−0.33	−11.7%
Breast	6	51.3%	−0.39	−0.02	−0.3%
Colorectal	6	27.4%	–	–	–
Prostate	2	7.8%	–	–	–

### Models accurately assess known cancer associations

**Figure 3:**
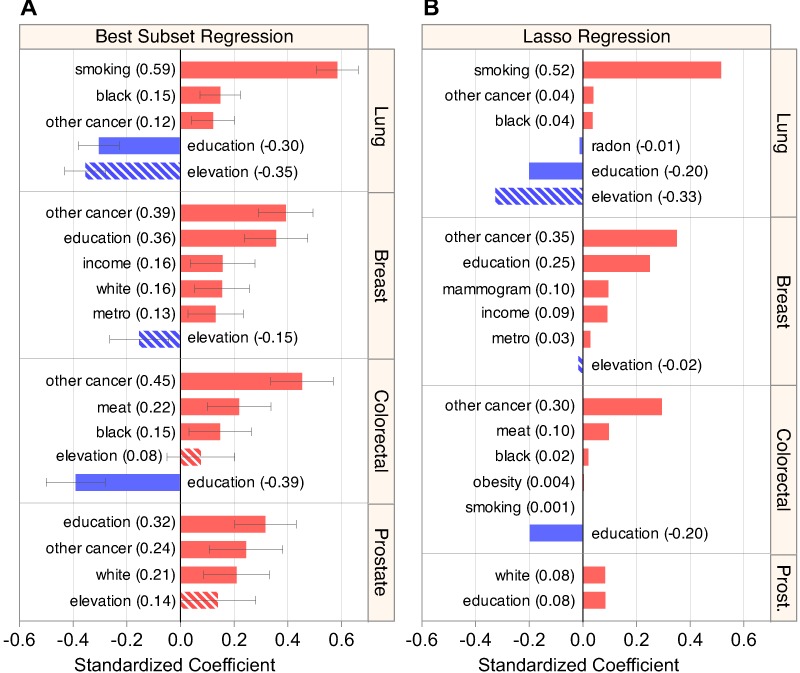
Regression models estimate elevation’s association while capturing known risk factors. (A) Summary of the predictors included in the optimal best subset model for each cancer (see Table S1 for more detail). (B) Summary of the models produced by lasso regression, displaying characteristic coefficient shrinkage. Both regression techniques produced similar sets of models that were sensible for lung, breast, and colorectal cancer. Elevation displayed a strong and consistent negative coefficient in lung cancer models.

Models produced for each cancer by best subset (Fig. 3A) and lasso (Fig. 3B) regression corresponded with the literature. The lasso (and best subset) models explained 67% (70%) of variation in lung cancer incidence, 51% (57%) in breast, 29% (34%) in colorectal, and 9% (19%) in prostate, (Tables 3 and 2) mirroring a previously described trend in fraction of risk attributable to modifiable factors for each of the four cancers (Danaei et al., 2005).

**Figure 4:**
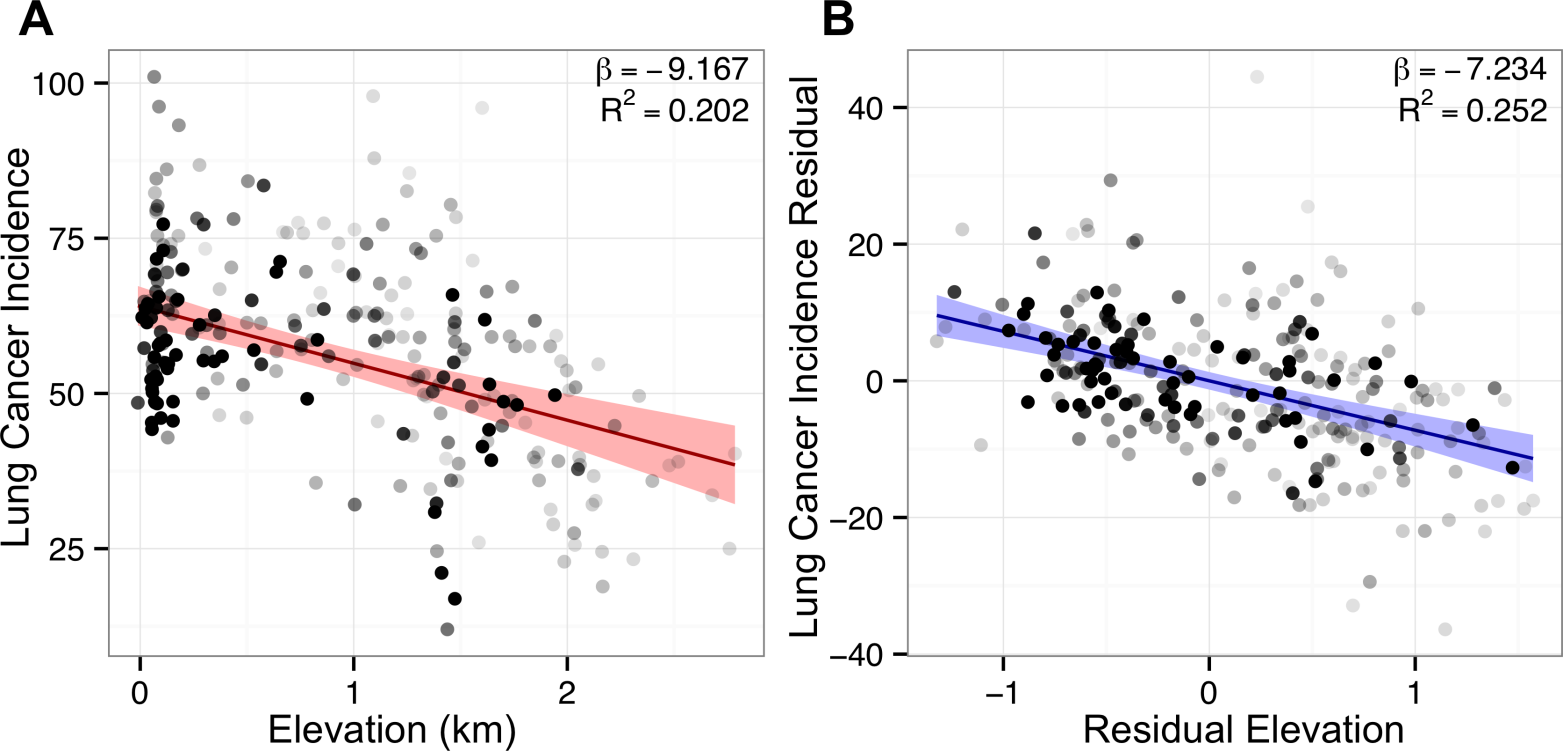
Adjustment for covariates sharpens lung cancer’s association with elevation. Points represent counties shaded by their regression weight based on population. Bivariate (red) and partial (blue) regression lines are displayed with 99% confidence bands. (A) Bivariate plot of county lung cancer incidence (age-adjusted per 100,000) and elevation (km). (B) Partial regression plot for elevation based on the optimal best subset lung model. Association sharpens after adjustment for covariates, illustrated by the tighter confidence band and higher *R*^2^ in the partial plot.

For lung cancer, both regression methods found previously characterized positive associations with smoking prevalence, percent of black residents (Greenlee et al., 2000, p. 10), and rate of other cancer (Ahlbom et al., 1997), as well as large negative associations with elevation and education. The lasso also found a small negative association with radon (*β_z_* = −0.01), attributable to radon’s strong positive correlation with elevation (Fig. 1). Including covariates sharpened the association between elevation and lung cancer, as evidenced by elevation’s higher partial *R*^2^ = 0.252 in the multivariate model versus the bivariate *R*^2^ = 0.202 (Fig. 4). Of note, both best subset and lasso regression attributed the two largest effect sizes to smoking (best subset: 0.59 and lasso: 0.52) and elevation (−0.35 and −0.33). The best subset model also found that smoking (*p* < 10^−35^) and elevation (*p* < 10^−16^) were the two most significant associations with lung cancer incidence (Table S1).

For breast cancer, the best subset regression model captured known positive associations with education and income (Devesa & Diamond, 1980), other cancer (Ahlbom et al., 1997), metropolitan status (Hall et al., 2005), and percent white (Greenlee et al., 2000, p. 10) (Howlader et al., 2014). The lasso corroborated the top three positive associations found by best subset regression, and added other known factors, mammography frequency and income (Devesa & Diamond, 1980), in place of percent white. As for lung and breast cancer, colorectal models were sensible, finding positive associations with other cancer, meat consumption (Norat et al., 2002), and percent of black residents (Greenlee et al., 2000, p. 10), as well as a negative association with education (Jemal et al., 2008)—all previously reported. In line with past ecological analyses (Danaei et al., 2005), prostate models were incomplete and inconclusive, failing to find the known positive association with percent black (Greenlee et al., 2000, p. 10). Overall, the plausible and well-fitting lung, breast, and colorectal models reflected our ability to recapitulate known associations and therefore characterize the prospective association of elevation with lung cancer.

### Elevation’s association with lung cancer is robust to stratification & subgrouping

**Figure 5:**
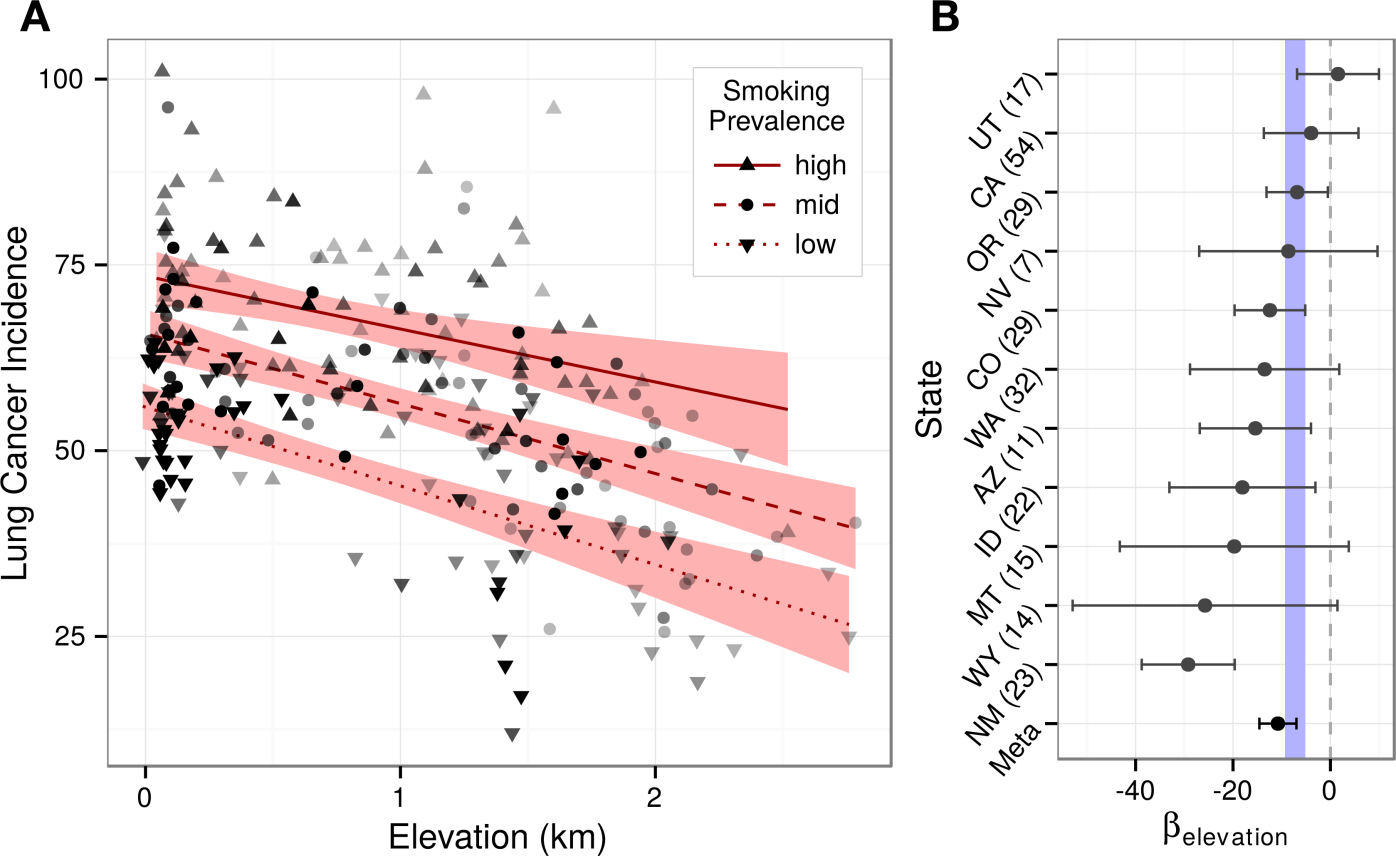
Elevation’s association with lung cancer is consistent across county strata. Stratification analyses provide no evidence for a smoking-elevation interaction or state-based confounding. (A) Lung cancer incidence (age-adjusted per 100,000) and elevation (km) are plotted for counties stratified into terciles by smoking prevalence. Counties were shaded by their regression weight, and a bivariate regression was fit for each stratum. The non-overlapping confidence bands (95%) illustrate the strong effect of smoking on lung cancer, while the approximately parallel slopes demonstrate the lack of an observable smoking-elevation interaction. (B) Lung cancer was regressed against elevation and smoking for each state. State-specific elevation coefficients are plotted with 95% confidence intervals. Ten of eleven states displayed negative coefficients. State-specific elevation coefficients were meta-analyzed. The resulting 99% confidence interval overlaps the interval from the optimal best subset model shown in blue. The number of counties within each state analysis is indicated in parentheses.

Given the respiratory intersection of oxygen inhalation and smoking, we asked whether elevation associated differently across smoking prevalences. Stratifying counties into smoking terciles, the strong effect of smoking on lung cancer was illustrated by the non-overlapping confidence bands (Fig. 5A). Tercile slopes were approximately parallel providing no evidence for an interaction between smoking and elevation. Corroborating the lack of interaction, an added smoking × elevation term was not significant (*p* = 0.47) when refitting the best subset model.

While focusing on the elevation-varying Western United States minimized the risk of regional confounding, possible differences in health policy or practice between higher and lower elevation states could still exist. State-specific models that accounted for smoking found negative elevation coefficients for ten of eleven states (Fig. 5B). Moreover, while small intrastate sample sizes created coefficient uncertainty, meta-analysis estimated that lung cancer incidence decreases by 10.8 99% CI [7.0–14.6] cases (per 100,000 individuals) per kilometer, matching the best subset regression confidence estimate of 7.2 99% CI [5.7–8.8] and indicating that state-based confounding did not underlie the negativity of the region-wide elevation association. To evaluate the consistency of state-specific elevation effects, we calculated *I*^2^, which equaled 71% 95% CI [35–90%]. Despite its uncertainty, the observed *I*^2^ suggests moderate to high heterogeneity across states (Higgins et al., 2003), which is unsurprising given the small sample sizes and limited covariate adjustment of the state-specific models.

**Figure 6:**
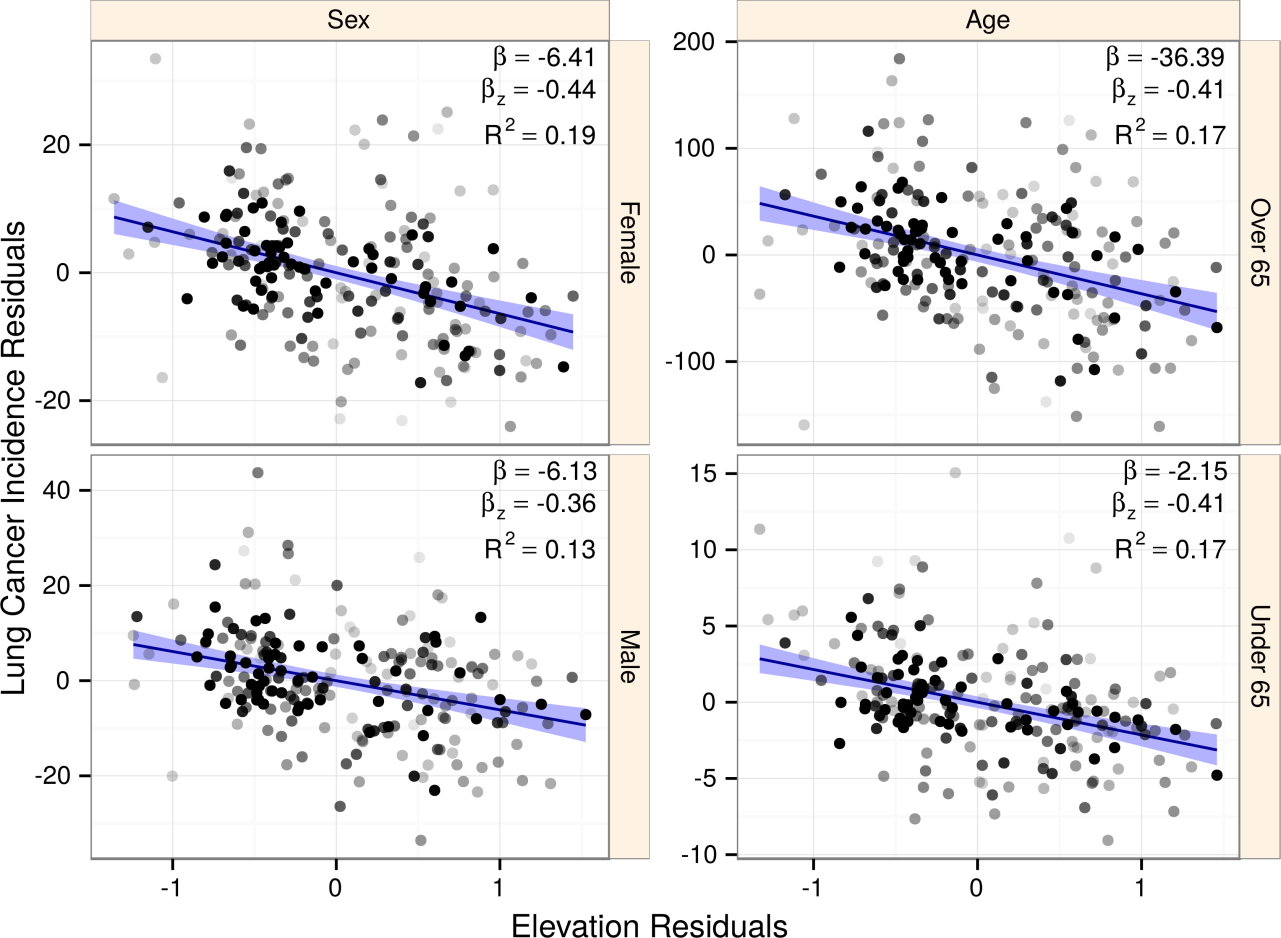
The association of elevation with lung cancer is consistent across population subgroups. Points represent counties shaded by their regression weight based on population. Partial regression plots are displayed for each subgroup with 99% confidence bands. Subgroups displayed similar strongly negative elevation associations, indicating that our findings were not the result of sex or age-based confounding. The elevation coefficient, standardized elevation coefficient, and partial *R*^2^ are listed for each partial regression.

Disparate habits and lifestyles exist across age groups and sexes. Using subgroup-specific lung cancer incidences, we refit the best subset model asking whether elevation association would change (Fig. 6). All four subgroupings showed significant, negative association with elevation: under 65 years old (*p* < 10^−12^), 65 and older (*p* < 10^−17^), males (*p* < 10^−14^), and females (*p* < 10^−18^). Standardized coefficients were large, ranging from −4.39 to −3.58. Subgroup confidence intervals all overlapped the confidence interval from the global model, showing no evidence for subgroup-specific effect mediation or confounding.

### Lung cancer associates with elevation over environmental correlates

**Figure 7:**
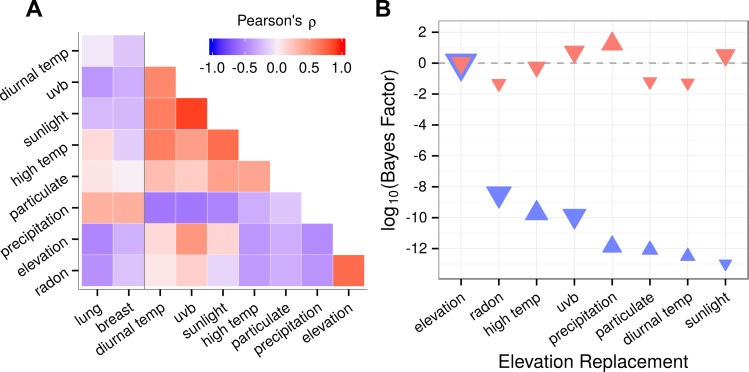
Environmental substitutes outperform elevation for breast but not lung cancer. (A) Environmental variables were strongly collinear. Correlation with elevation was high, hence many environmental variables also covaried with lung and breast cancer. (B) To test whether elevation-association with lung and breast cancer was direct or indirect, we substituted each environmental correlate in place of elevation during best subset selection for each cancer. The optimal model for each elevation-replacement was compared to the unreplaced model by approximating a Bayes factor (*K*) from the change in BIC. The Bayes factor indicates the odds that the replacement is superior, thus *K* > 1 favors the substitution while *K* < 1 provides evidence against. Since the elevation model was compared to itself, Δ*BIC* = 0 and *K* = 1 (log_10_*K* = 0). The standardized coefficient for each environmental predictor is represented by a triangle, where size is scaled to the magnitude and orientation indicates the sign (upwards for positive). For breast cancer (red), three substitutions increased likelihood suggesting that any association observed with elevation was indirect. For lung cancer (blue), substituting elevation produced models that were many orders of magnitude less likely, suggesting that the elevation association was direct.

Rising elevation leads to lower atmospheric pressure, which helps drive a repertoire of interconnected climatic changes, including perturbations in sun exposure, temperature, and precipitation. This trend was apparent in our data, as many environmental variables correlated with elevation, and thus also with lung and breast cancer (Fig. 7A). If either cancer’s elevation association was indirect of atmospheric pressure but rather a product of secondary climatic changes, we expected that environmental correlates could outperform elevation in best subset regression. For example, vitamin D synthesis is stimulated by sunlight and UV exposure (Gilchrest, 2008). The hormonally active form of vitamin D, calcitriol, potentially possesses anti-cancer properties (Krishnan & Feldman, 2011). Sunlight and UVB exposure correlate positively with elevation in our data. Hence, a reasonable proposition would be that increased vitamin D synthesis is driving elevation’s association with lower cancer rates (as posited by Hayes, 2010) and that replacing elevation with UVB would improve model likelihood.

We approximated the likelihood (as a Bayes factor, *K*) that any correlated environmental variable could replace elevation in our models of lung and breast cancer (Fig. 7B). Remarkably, for lung cancer, all of the variables tested produced models between 10^8^ and 10^13^ times less likely than the elevation-including model. Among these, fine particulate matter was over one trillion times (*K* < 10^−12^) less likely than elevation. In contrast, breast cancer was more effectively explained by environmental correlates or secondary climatic changes, such as precipitation, UVB, and sunlight, than by elevation. For example, precipitation was 17 times more likely than elevation. Together, these results indicated that lung cancer but not breast cancer was directly associated with atmospheric pressure.

### Radon and UVB associations with lung cancer confounded by elevation

As a consequence of elevation’s numerous environmental correlates, we speculated that previous ecological studies of lung cancer may have fallen prey to uncontrolled elevation confounding. Radon (*ρ* = 0.71) and UVB (*ρ* = 0.50) correlated highly with elevation in our data (Fig. 7A) and both had previously been reported to associate negatively with lung cancer (Cohen, 1995; Hayes, 2010). To test for confounding, we fit three models of lung cancer incidence with either radon or UVB exposure plus the following predictors: (1) smoking alone; (2) smoking and elevation; and (3) the predictors from the optimal best subset model. Similar to previous studies, model 1, which excluded elevation, identified a negative association for both radon (*p* < 10^−14^, one-tailed) and UVB (*p* < 10^−3^). However, models 2 and 3, which both included elevation, erased these associations (Table 4), indicating high potential for erroneous lung cancer associations when not accounting for elevation.

**Table 4:** Confounding effect of elevation on radon and UVB lung cancer associations. Lung cancer incidence models for were fit for three sets of predictors: (model 1) radon/UVB and smoking; (model 2) radon/UVB, smoking, and elevation; and (model 3) radon/UVB and the optimal best subset predictors. The standardized UVB/radon coefficient (*β_z_*) [95% confidence interval] and UVB/radon *p*-value for coefficient negativity are reported.

	Radon		UVB	
Model	*β_z_*	*p*-value	*β_z_*	*p*-value
1	−0.36 [−0.44, −0.28]	1.90× 10^−15^	−0.18 [−0.28, −0.08]	3.08× 10^−04^
2	−0.07 [−0.18, 0.04]	0.120	0.05 [−0.04, 0.15]	0.864
3	−0.03 [−0.13, 0.07]	0.308	−0.01 [−0.10, 0.09]	0.453

## Discussion

We attributed a decrease of 25.2 99% CI [18.0–32.4] lung cancer cases per 100,000 individuals to the range of elevation of counties of the Western United States, equating to approximately 44% of the mean incidence (56.8). Were the entire United States situated at the elevation of San Juan County, CO (3,473 m), we estimate 65,496 99% CI [46,855–84,136] fewer new lung cancer cases would arise per year (*ceteris paribus* and assuming 2000-census county-populations). The causal factor behind the association appears to play a notable role in lung carcinogenesis, worthy of consideration by researchers, health providers, and the general public.

Prior to covariate adjustment, elevation correlated negatively with lung, breast, and colorectal cancer. Adjusting for demographic and risk factors using best subset and lasso regression, we produced sensible models for each cancer that captured known risk factors. Elevation’s negative association with colorectal cancer disappeared with adjustment for demographics, while association with breast cancer proved to be minimal and could be better represented by several other environmental variables. In contrast, lung cancer’s negative association with elevation was sharpened following multiple regression, failed to be captured by any other environmental variables, and had a remarkably strong effect size and significance. Lung-elevation association was robust to county stratification by smoking and state, as well as to population subgrouping by age and sex.

In summary, lung cancer associated with elevation over oxygen-independent environmental factors, and likewise elevation associated with lung cancer but not with non-respiratory cancers. Together these points provide substantial evidence for an inversely-linked inhaled carcinogen tied directly to elevation. Viewing our findings through the lens of the literature, atmospheric oxygen emerges as the most probable culprit.

### Confounding effect of elevation

Since elevation commonly covaries and its effect size on lung cancer is large, the potential for confounding is high. We identified two reported lung cancer associations—radon (Cohen, 1995) and UVB (Hayes, 2010)—that we attributed wholly to elevation in our analyses. Previously, Lagarde & Pershagen (1999) implicated ecological fallacy in the appearance of weak inverse associations between radon and lung cancer in Sweden. However, the plausibility of ecological fallacy resulting in the strong inverse association Cohen (1995) observed across American counties is less clear. Alternatively, Van Pelt (2003) attributed “some, but not all” of the Cohen (1995) radon association to elevation. Follow-up correspondences by each author revolved around the difficulty in assigning the effect wholly to elevation or radon when both of these highly-correlated predictors remained significant (Cohen, 2004; Van Pelt, 2004). We believe that our data quality improvements, including county-specific smoking prevalences and population-weighted elevations, were responsible for wholly attributing the effect to elevation.

Studies where lung cancer is incorporated as a predictor rather than outcome may also be susceptible to confounding. Since the collection and availability of tobacco data has historically lagged behind lung cancer data, lung cancer rates have often been adopted as a proxy for smoking prevalence (Peto et al., 1992). Ezzati et al. (2012) evaluated whether elevation was associated with several mortality outcomes across US counties. Their study relied on “lung cancer as the indicator of accumulated population exposure to smoking. This adjustment for lung cancer in multivariable regressions may have over-adjusted, if altitude has a beneficial effect on lung cancer.”

The confounding potential of elevation extends to any analysis of lung cancer across an elevation-varying region. To protect against this uncontrolled confounding, we urge future lung cancer studies to strongly consider adjusting for elevation. Fortunately, elevation is well-documented across the globe, and many existing epidemiological datasets contain locality information.

### Limitations & future directions

Cross-sectional study designs are susceptible to uncontrolled confounding where associations arise due to an unmeasured confounding factor. We designed the study to minimize this risk by focusing on a homogeneous and elevation-varying region, filtering error-prone counties, including established covariates, and performing multiple stratifications and subgroupings. Furthermore, while environmental correlates of elevation represented likely confounders, all seven pervasive environmental factors we investigated could not replace elevation in models of lung cancer. Therefore, our findings gave no indication of uncontrolled confounding for lung cancer. As relevant data becomes available, follow-up across different regions will provide additional assessment of uncontrolled confounding.

Since we evaluated counties rather than individuals, ecological fallacy was also a concern. However, several of our methods and findings limit this possibility. By focusing on US counties, the smallest population grouping with systematic data available for the target region, we inherently reduced the risk of ecological fallacy. Moreover, further increases in group specificity through population subgrouping did not alter the elevation-lung association. Additionally, we reason that an exposure affecting only a portion of the population must confer a very large risk to produce the strong association observed. We find it unlikely that an extremely damaging risk factor on the individual level, such as smoking, would have evaded detection until now. Therefore, we speculate the causal factor is likely mild in carcinogenicity but universal in exposure and thus amenable to translation from the individual to population level. Follow-up biological and experimental analyses will be critical to understanding the causal factor and potential mechanisms underlying the observed elevation association. If future research confirms oxygen-driven tumorigenesis in the human lung, the present study will join the substantial list of ecological analyses that spurred new insights into cancer etiology (Pearce, 2000).

### Open data

This study was made possible by excellent county level resources, many of which have only recently become available. While the trend towards available, accessible, and reusable data is encouraging, barriers still remain. For example, the 2006–2010 State Cancer Profiles release is missing incidence for two thirds of the counties in Washington due to “state legislation and regulations which prohibit the release of county level data to outside entities.” As the world comes online and the number of people with access to informatics tools expands, we see the spread of open data as a vital catalyst for progress.

## Supplemental Information

10.7717/peerj.705/supp-1 Figure S1 Quality control: selecting exclusion thresholds for counties with high Native American and immigration percentages We suspected misestimated cancer rates for counties with a high Native American percentage and a poor ability of predictors to assess cancer-risk exposure for counties with high immigration rates. To examine whether these counties were problematic, we created a general model of cancer incidence by regressing all-site cancer incidence against eight demographic and health-related covariates (metro, white, black, education, income, obesity, percent male, and smoking). Elevation was not included in the model to prevent opportunistic threshold selection. The regression was fit on Western-US counties with populations of at least 10,000. Absolute residuals are plotted against percent Native American and the 5-year immigration rate for each county (shaded by their population-based regression weight). Loess curves (displayed in blue with 95% confidence bands) indicate that predicted incidence diverged from reported incidence for both native and immigration-rich counties. Exclusion thresholds were selected, above which counties were filtered (red background), corresponding to the values where absolute residuals began trending higher.

10.7717/peerj.705/supp-2 Table S1 Optimal best subset regression models Coefficient estimates from are displayed in unstandardized (*β*) and standardized (*β_z_*) forms followed by the confidence interval. The two-tailed coefficient *p*-value is reported.

10.7717/peerj.705/supp-3 Dataset S1 County-level dataset Tab delimited data collected for US counties. Missing values are blank. Source-reported 95% confidence intervals have ‘lower’ and ‘upper’ appended to the corresponding variable name.
