# Supplementary figures and images for: Lung cancer incidence decreases with elevation: evidence for oxygen as an inhaled carcinogen
Source: PeerJ. 2015 Jan 13;3:e705. doi: 10.7717/peerj.705 (PMC4304851; doi:10.7717/peerj.705)

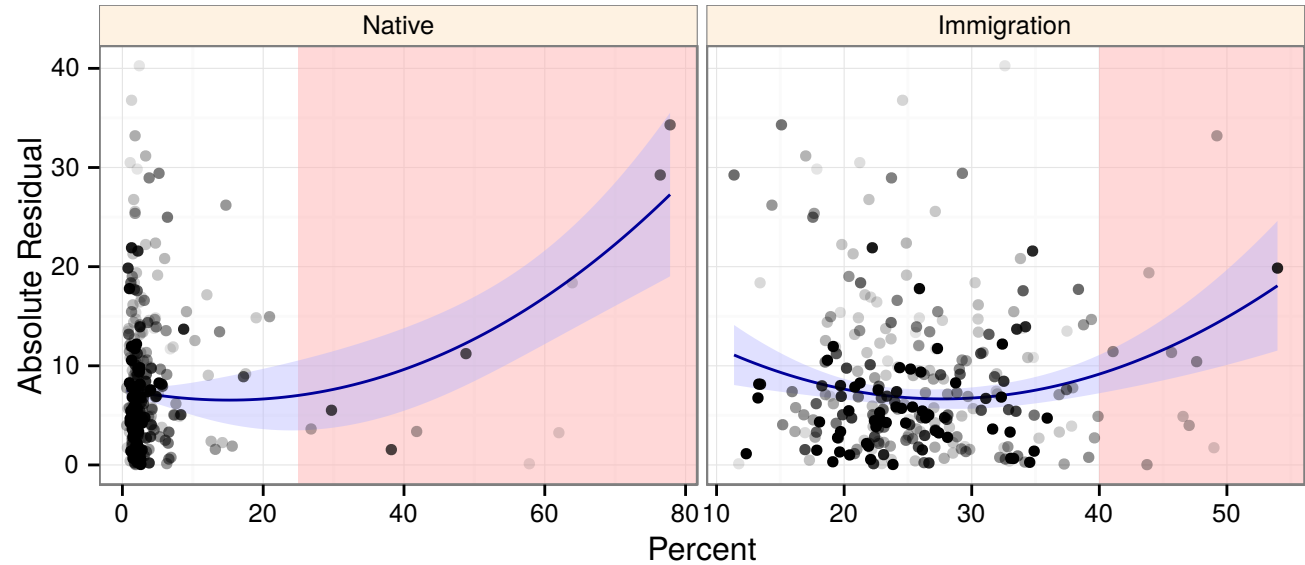

Supplement: Figure S1 — We suspected misestimated cancer rates for counties with a high Native American percentage and a poor ability of predictors to assess cancer-risk exposure for counties with high immigration rates. To examine whether these counties were problematic, we created a general model of cancer incidence by regressing all-site cancer incidence against eight demographic and health-related covariates (metro, white, black, education, income, obesity, percent male, and smoking). Elevation was not included in the model to prevent opportunistic threshold selection. The regression was fit on Western-US counties with populations of at least 10,000. Absolute residuals are plotted against percent Native American and the 5-year immigration rate for each county (shaded by their population-based regression weight). Loess curves (displayed in blue with 95% confidence bands) indicate that predicted incidence diverged from reported incidence for both native and immigration-rich counties. Exclusion thresholds were selected, above which counties were filtered (red background), corresponding to the values where absolute residuals began trending higher. [file peerj-03-705-s001.pdf]
