# Supplementary material for: Lung cancer incidence decreases with elevation: evidence for oxygen as an inhaled carcinogen
Source: PeerJ. 2015 Jan 13;3:e705. doi: 10.7717/peerj.705 (PMC4304851; doi:10.7717/peerj.705)
Supplement: Table S1 — Coefficient estimates from are displayed in unstandardized (β) and standardized (βz) forms followed by the confidence interval. The two-tailed coefficient p-value is reported. [file peerj-03-705-s002.pdf]

cancer	predictor	β	β_z	p-value
lung	smoking	1.44 [1.25, 1.63]	0.59 [0.51, 0.66]	3.46×10^{-36}
lung	elevation	-7.23 [-8.80, -5.67]	-0.35 [-0.43, -0.28]	2.69×10^{-17}
lung	education	-0.43 [-0.54, -0.33]	-0.30 [-0.38, -0.23]	1.08×10^{-13}
lung	black	0.67 [0.33, 1.01]	0.15 [0.07, 0.22]	1.24×10^{-04}
lung	other cancer	0.05 [0.02, 0.08]	0.12 [0.04, 0.20]	3.04×10^{-03}
lung	(Intercept)	-14.85 [-28.21, -1.50]	0.00 [-0.07, 0.07]	2.94×10^{-02}
breast	other cancer	0.22 [0.16, 0.28]	0.39 [0.29, 0.49]	7.98×10^{-13}
breast	education	0.58 [0.39, 0.78]	0.36 [0.24, 0.47]	8.45×10^{-09}
breast	income	0.22 [0.05, 0.39]	0.16 [0.04, 0.28]	1.08×10^{-02}
breast	white	0.21 [0.07, 0.35]	0.16 [0.05, 0.26]	2.95×10^{-03}
breast	elevation	-3.63 [-6.23, -1.03]	-0.15 [-0.26, -0.04]	6.44×10^{-03}
breast	metro	4.58 [0.98, 8.19]	0.13 [0.03, 0.23]	1.29×10^{-02}
breast	(Intercept)	14.45 [-3.29, 32.18]	0.00 [-0.08, 0.08]	1.10×10^{-01}
colorectal	other cancer	0.07 [0.05, 0.09]	0.45 [0.34, 0.57]	4.23×10^{-13}
colorectal	education	-0.23 [-0.30, -0.17]	-0.39 [-0.50, -0.28]	2.51×10^{-11}
colorectal	meat	0.09 [0.04, 0.14]	0.22 [0.10, 0.34]	3.36×10^{-04}
colorectal	black	0.28 [0.06, 0.49]	0.15 [0.03, 0.26]	1.27×10^{-02}
colorectal	elevation	0.65 [-0.42, 1.72]	0.08 [-0.05, 0.20]	2.34×10^{-01}
colorectal	(Intercept)	12.01 [3.74, 20.29]	0.00 [-0.10, 0.10]	4.62×10^{-03}
prostate	education	0.75 [0.48, 1.02]	0.32 [0.20, 0.43]	1.25×10^{-07}
prostate	other cancer	0.13 [0.06, 0.21]	0.24 [0.11, 0.38]	5.01×10^{-04}
prostate	white	0.42 [0.17, 0.66]	0.21 [0.09, 0.33]	8.60×10^{-04}
prostate	elevation	4.71 [-0.05, 9.46]	0.14 [-0.00, 0.28]	5.23×10^{-02}
prostate	(Intercept)	43.46 [11.65, 75.28]	0.00 [-0.11, 0.11]	7.61×10^{-03}
